# Inaugural meeting of Global Outbreak Alert and Response Network partners from Australia

**DOI:** 10.5365/wpsar.2024.15.5.1137

**Published:** 2024-03-11

**Authors:** Cassandra Dearing, Kieh Christopherson, Peta-Anne Zimmerman, Sharon Salmon

**Affiliations:** a World Health Organization Regional Office for the Western Pacific, Manila, Philippines.; b Indo-Pacific Centre for Health Security, Department of Foreign Affairs and Trade, Canberra, Australia.; c Australasian College for Infection Prevention and Control, Hobart, Tasmania, Australia.; d Griffith University, Gold Coast, Queensland, Australia.; e Collaborative for the Advancement of Infection Prevention and Control, Gold Coast, Queensland, Australia.; f UNSW Medicine, School of Public Health and Community Medicine, University of New South Wales, Sydney, New South Wales, Australia.

Established in April 2000, the Global Outbreak Alert and Response Network (GOARN) is a mechanism to allow partners, including technical institutions and networks around the world, to work together to assist World Health Organization (WHO) Member States in responding to public health emergencies. (*1*) As of February 2024, GOARN has more than 300 partners. (*2*) Following the GOARN Global Meeting of Partners in 2021, the GOARN Steering Committee adopted the first GOARN strategy 2022–2026. (*3*) The strategy builds upon its original founding principles and commits the Network to four strategic themes, including “applying a community-centred approach.”

In May 2023, a workshop was held with GOARN Steering Committee members and selected partners to develop a strategy implementation plan that operationalized GOARN’s aim of working closer to communities. Strategic grouping was agreed to as a priority to enable partners to convene in smaller groups based on mutual interests. Groupings can be based on common interests relevant to GOARN’s strategic themes, such as a public health technical area, geography (national, subregional, regional), language (for example, a working coalition of Francophone or Portuguese-speaking countries sharing materials and resources) or other strategic alignments. These groups can actively engage partners within a large global network, enhance information sharing and communication, and promote new opportunities for partners who are less engaged in Network activities and deployments.

A strategic grouping of GOARN focal points from institutional partners in Australia was set up, and an inaugural meeting was convened virtually on 8 November 2023.

Australia hosts 25 out of 76 (33%) GOARN partners in the Western Pacific Region. Since GOARN’s establishment in 2000, 18 of these 25 partners have made at least one offer to deploy, with 15 partners deploying an expert at least once. A total of 71 individuals have been deployed to 94 missions for 19 global operations. (*2*)

The purpose of the meeting was to gather and connect GOARN focal points from Australia to strengthen collaboration between partners to improve Network participation and connectivity among partners, share experiences, and determine interest in remaining connected.

The meeting was hosted virtually by the WHO Regional Office for the Western Pacific. The agenda was designed with partners who attended the strategy implementation workshop: Australian National University, Australasian College for Infection Prevention and Control and Australia’s Indo-Pacific Centre for Health Security.

The 2-hour programme consisted of four sessions and was attended by 36 participants, including 34 GOARN focal points representing 18 of the 25 partners from Australia (**Box 1**). All participants delivered a self-introduction including a description of the key technical areas of their institution.

**Box 1:**
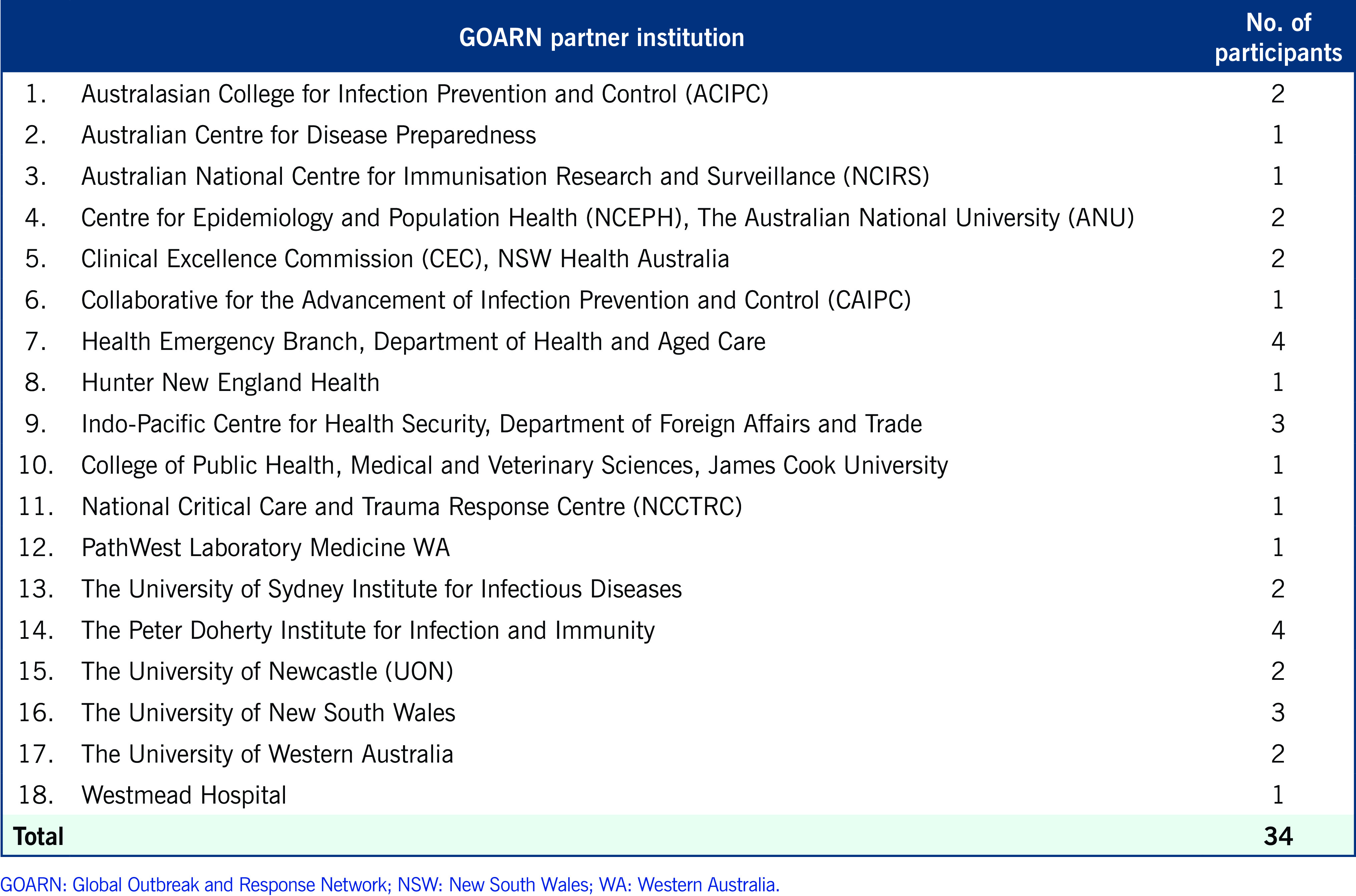
Participants of the inaugural meeting of GOARN partners in Australia, 8 November 2023

[**insert** Box 1]

The first session oriented participants to GOARN’s strategy, deployment process, areas of work and recent activities in the WHO Western Pacific Region.

The second session comprised a moderated panel discussion with three panellists representing a university, government agency and technical network. They explained methods for sharing GOARN communications within the institution and efforts to improve and streamline dissemination, including the use of external instant messaging platforms to enable fast delivery of GOARN requests for assistance. Panellists shared ways of identifying individuals from their institution who are suitable for deployment and ways of recording interest such as through an informal roster of experts.

During the third session, three participants presented their institution’s involvement in GOARN activities. Speakers shared experiences participating, delivering and hosting GOARN trainings, conducting operational research, and opportunities for potential collaboration and developing new initiatives to support GOARN activities including deployment mentorship models. Speakers shared personal accounts highlighting how participation in the GOARN capacity strengthening and training programme (*4*) enhanced personal preparedness to deploy to an international outbreak response mission.

The final session provided a space for virtual interaction among all participants using the interactive presentation tool Mentimeter (Stockholm, Sweden) to understand how partners currently engage with GOARN and how they would engage in the future. The majority of partners reported engagement with the Network through the GOARN Technical Officer based in the WHO Regional Office for the Western Pacific and through accessing information on the GOARN Knowledge Platform. (*3*) Partners expressed interest in meeting quarterly or every 6 months, with a preference to meet face-to-face with virtual attendance as an option. Conferences were identified as a potential option to host future meetings.

Participants were highly satisfied with this initial meeting and used the opportunity to reach out to each other through the virtual chat function. Partners plan to continue meeting and strengthening cross-collaboration and engagement within GOARN, which will improve preparedness and response to imminent future public health emergencies. Partners have proposed alternating the meeting chair to enhance shared leadership of this group.

Regional or subregional groupings can help put GOARN’s objectives and activities in the context of partners’ own country, subregion or region, and identify and mobilize resources to increase participation and engagement among partners to implement GOARN activities closer to communities.
